# Synergistic Phenolic Compounds in Medicinal Plant Extracts: Enhanced Furin Protease Inhibition via Solvent-Specific Extraction from Lamiaceae and Asteraceae Families

**DOI:** 10.3390/molecules30173450

**Published:** 2025-08-22

**Authors:** Fatime Betül Üzer, Nazlı Helvacı, Mahfuz Elmastaş

**Affiliations:** Department of Phytotherapy, University of Health Sciences, Selimiye Complex, Selimiye Neighborhood, Atolyeler Street No: 4, Uskudar, 34668 Istanbul, Turkey; tokazitaki.55@gmail.com (N.H.); mahfuz.elmastas@sbu.edu.tr (M.E.)

**Keywords:** medicinal plant extract, furin protease, phenolic compounds, combination index, synergy

## Abstract

This study aimed to evaluate the inhibitory potential of phenolic-rich extracts from selected medicinal plants belonging to the *Lamiaceae* and *Asteraceae* families against the furin protease enzyme, a key target in viral and oncogenic pathways. Extracts of *Origanum vulgare*, *Thymus vulgaris*, *Mentha piperita*, *Mentha spicata*, *Salvia officinalis*, and *Silybum marianum* were prepared using hexane, chloroform, and ethyl acetate. Phenolic compounds were quantified using High-Performance Liquid Chromatography (HPLC). Furin inhibition was assessed spectrophotometrically and analyzed statistically with multivariate approaches. The chloroform extract of *Origanum vulgare* exhibited 97.44 ± 0.12% inhibition, while the ethyl acetate extract of *Mentha spicata* showed 97.44 ± 0.08% inhibition. Epicatechin and rutin displayed significant synergistic effects, while naringenin negatively affected inhibition (*p* < 0.05). Solvent polarity significantly influenced phenolic diversity and biological activity, with ternary combinations showing 33% higher inhibition than single compounds. These findings highlight phenolic blends as promising natural furin inhibitors, with chloroform being optimal for broad-spectrum extraction.

## 1. Introduction

Furin is a pivotal protease enzyme in humans, localized in the Golgi apparatus and on the cell surfaces of numerous organisms, including mammals. It exists in both membrane-bound and secreted forms. Under normal physiological conditions, furin cleaves and activates over 100 proprotein and peptide substrates. Independently of other risk factors, furin has been associated with increased risks of diabetes, hyperinsulinemia, hypertension, hyperlipidemia, obesity, and all-cause mortality [1,2].

Beyond its essential role in Severe Acute Respiratory Syndrome Coronavirus 2 (SARS-CoV-2) cellular entry and replication, furin also activates multiple peptides that are critical to both normal physiology and the pathogenesis of COVID-19. Numerous bacterial and viral pathogens utilize furin protease for host cell entry or toxin activation. For instance, *Bacillus anthracis* (the anthrax pathogen) requires furin for cellular invasion [3], and the α-toxin of *Clostridium septicum* (the agent of gas gangrene) is activated by furin [4]. Other furin-dependent pathogens include *Pseudomonas aeruginosa* exotoxin A, Shiga toxin, and diphtheria toxin [5]. Several viruses, including paramyxoviruses, HIV-1, Ebola virus, and herpes simplex virus-1, also depend on furin-mediated proteolytic activation [6,7,8,9,10,11]. Furin modulates the renin–angiotensin–aldosterone system (RAAS) through activation of the pro-renin receptor, thereby facilitating vasoconstrictive angiotensin production [3,4]. It plays crucial roles in coagulopathy via the processing of von Willebrand factor and factor VIII, with furin being indispensable for the activation of factor VIII [12,13]. Notably, furin expression is upregulated under hypoxic conditions.

As a proprotein convertase, furin is implicated in tumorigenesis and cancer progression. Aberrant furin expression or activity has been reported in colon carcinoma, rhabdomyosarcoma, and head and neck, lung, skin, and brain tumors. In these contexts, furin promotes proliferation, angiogenesis, migration, invasion, and metastasis [14].

Given its multifunctional roles in pathology, furin represents a prime therapeutic target. While chronic inhibition of furin may lead to adverse effects, short-to-medium-term inhibition could provide an effective strategy against furin-dependent infections. Currently known furin inhibitors include macromolecules such as α_1_-antitrypsin Portland and inter-α-inhibitor protein; reactive small molecules such as chloromethylketone and aminomethylketone derivatives; peptide-based inhibitors including poly-arginine variants; and non-peptidic compounds such as amidinohydrazones, metal chelates, and andrographolide. However, macromolecules often suffer from poor stability, and chloromethylketones exhibit cytotoxicity [15,16]. Non-peptidic small molecules present better potential for drug development, although few highly effective inhibitors have been identified. Notably, andrographolide from *Andrographis paniculata* has demonstrated only weak furin inhibition [17].

The growing health concerns surrounding synthetic compounds have revitalized global interest in plant-derived therapeutics, particularly within the pharmaceutical, cosmetic, and food industries. Phytotherapy has emerged as a multidisciplinary field focusing on the study of plant secondary metabolites. Current research priorities in this area include the identification and characterization of bioactive compounds, the screening of structurally related phytochemicals, pharmacological evaluation of biological activities, and the sustainable cultivation of medicinal plants.

Ethnobotanical studies have confirmed the therapeutic use of numerous species from the Turkish flora [18,19,20,21]. However, effective integration of these plants into modern medicine requires the standardization of active compounds, the optimization of formulation stability, mechanistic elucidation of potential synergistic effects, and clinical validation.

In this study, we investigated the furin inhibitory potential of selected medicinal plants using molecular docking, in silico predictions, and in vitro analyses. Our focus was on ethnomedicinal species from Turkey, particularly those belonging to the *Lamiaceae* family (*Thymus vulgaris*, *Origanum vulgare*, *Mentha piperita, Mentha spicata*, and *Salvia officinalis*) and the *Asteraceae* family (*Silybum marianum*). These species were selected based on their secondary metabolite profiles, which included compounds predicted to inhibit furin activity.

The *Lamiaceae* family is one of the most therapeutically important botanical families. The medicinal efficacy of its members is primarily attributed to their high essential oil content. As one of the largest dicot families, *Lamiaceae* includes a wide range of aromatic shrubs characterized by hairy stems and ovate leaves that are typically green or purple and up to 5 cm in length. Notable genera include Origanum, Mentha, Thymus, and Lavandula. Reported pharmacological activities in this family include antidiabetic, anticancer, antimicrobial, and hepatoprotective effects [20,21].

The *Asteraceae* family is globally distributed and morphologically diverse, encompassing trees, shrubs, and herbs that often bear small, clustered florets. Traditional Turkish medicine utilizes many *Asteraceae* species due to their rich content of essential oils, phenolic acids, flavonoids, saponins, and inulin-type prebiotics. These phytochemicals contribute to a wide spectrum of biological activities, including antioxidant, anti-inflammatory, antimicrobial, and wound-healing properties, often through mechanisms involving free radical scavenging [22,23].

Using solvents of varying polarity—hexane, chloroform, and ethyl acetate—we prepared plant extracts and evaluated their furin inhibitory activities through in vitro assays.

## 2. Results

### Findings Related to Phytochemical Studies

The highest levels of furin inhibition were observed in *Origanum vulgare* (chloroform extract), *Mentha spicata* (ethyl acetate extract), and *Salvia officinalis* (chloroform extract), each exhibiting over 97% inhibition. Among these, *Origanum vulgare* (chloroform) showed 97.44 ± 0.12% inhibition (ANOVA: F (2, 9) = 45.6, *p* < 0.001), which was attributed to its rich phenolic profile, primarily consisting of epicatechin (2.277 mg/g extract) and a small amount of rutin (0.051 mg/g extract). *Mentha spicata* (ethyl acetate) demonstrated an almost identical inhibition rate of 97.44 ± 0.08% (ANOVA: F (2, 9) = 38.9, *p* < 0.001), with notable levels of quercetin (0.07 mg/g), rutin (1.555 mg/g), cinnamic acid (2.509 mg/g), and naringenin (5.315 mg/L), suggesting that the synergistic presence of multiple active compounds may contribute to its high inhibitory potential.

Similarly, *Salvia officinalis* (chloroform) extract recorded 97.44 ± 0.10% inhibition (ANOVA: F (2, 9) = 41.7, *p* < 0.001). Though its phenolic content was relatively simpler, consisting mainly of rutin (0.225 mg/g) and cinnamic acid (0.021 mg/g), the high inhibition level suggests that even low concentrations of certain phenolics may exert significant furin inhibitory effects when extracted with an efficient solvent system.

In contrast, the lowest furin inhibition activities were observed in *Thymus vulgaris* (ethyl acetate) at 56.41 ± 1.2% and *Mentha piperita* (chloroform) at 66.66 ± 0.9%, possibly due to their limited content of key phenolic compounds or the absence of synergistically interacting molecules (Table 1).

A statistically significant difference was found among phenolic compound combination types (F (3, 9) = 28.4, *p* < 0.001). Triple and quadruple combinations resulted in significantly higher inhibition than single or double combinations (*p* < 0.01), while no significant difference was observed between single and double combinations (*p* > 0.05). Each additional compound in the mixture was associated with an average increase of 8.9% in inhibition. Among the individual compounds, epicatechin showed a positive and significant effect on furin inhibition (β = +12.3, *p* < 0.01), naringenin exhibited a negative and significant effect (β = −8.7, *p* < 0.05), and quercetin had no statistically significant association with inhibition (*p* > 0.05) (Table 2).

Notably, the combination of rutin and epicatechin displayed a synergistic effect, significantly enhancing the level of inhibition (β = +9.1, *p* < 0.05). An increase in the number of phenolic components was positively correlated with inhibition rates, supported by a strong and significant Pearson correlation (r = +0.89, *p* < 0.01). On average, triple combinations resulted in 33% higher inhibition compared to single compounds (*p* < 0.001). In general, the addition of each phenolic compound increased inhibition by 8.9 ± 1.2% on average (*p* < 0.01) (Table 3).

The relationship between phenolic compound diversity and inhibition percentage was examined across different solvents using Pearson’s correlation analysis, which revealed a strong positive correlation (r = +0.81, *p* < 0.001). As phenolic diversity increased, furin inhibition also significantly increased. The average number of phenolic compounds identified per solvent was as follows:

Chloroform: 4.2 ± 0.8; ethyl acetate: 3.5 ± 0.6; hexane: 1.3 ± 0.4.

Among solvents, chloroform was the most effective in extracting epicatechin (2.277 mg/L, *p* < 0.01), while ethyl acetate proved optimal for extracting polar compounds such as cinnamic acid (2.509 mg/L). In contrast, hexane selectively extracted non-polar compounds, such as quercetin (range: 0.233–2.71 mg/L). The polarity of the solvent used during the extraction process was found to significantly influence both the composition of extracted phenolic compounds and the corresponding furin inhibitory activity. Chloroform emerged as the most effective solvent, demonstrating a broad-spectrum capacity to extract diverse phenolic constituents and yielding the highest biological activity. In contrast, ethyl acetate and hexane were observed to be more limited in their extraction profiles but effective in isolating specific phenolic compounds (Table 4).

## 3. Discussion

Furin is a critical protease that facilitates the maturation of numerous proteins both inside and outside the cell. This enzyme specifically recognizes polybasic amino acid sequences, cleaving and activating proteins. The importance of furin lies in its role in physiological processes as well as its ability to enhance the virulence of pathogens. Furin mediates the maturation of essential proteins such as insulin-like growth factors (IGFs), platelet-derived growth factor (PDGF), and vascular endothelial growth factor (VEGF). These factors play crucial roles in cell growth, tissue repair, and immune system regulation. The activation of cell surface proteins, including integrins and receptors, is necessary for cellular interactions, and furin ensures their functional maturation. Deletion of the furin gene in mice has been reported to cause embryonic lethality [13], highlighting its vital role in cardiovascular and organ development.

Many viruses exploit furin for host cell entry, including the following:

- SARS-CoV-2: Cleavage of the “RRAR” motif in the spike protein by furin enhances viral binding to the ACE2 receptor and cell entry.
- HIV: Processing of the Env protein by furin is essential for viral infectivity.
- Highly Pathogenic Influenza Viruses: Furin-mediated cleavage of hemagglutinin (HA) facilitates systemic infections.

Furin also activates bacterial toxins such as diphtheria toxin and Shiga toxin, increasing their cytotoxic effects. Additionally, furin promotes tumor progression by activating proteins like TGF-β and MMPs. The overexpression of furin may accelerate cancer metastasis. Furin modulates immune responses by processing cytokines such as IFN-γ and TGF-β [13], which are critical for defense against infections and prevention of autoimmune diseases.

An “RRAR” motif, formed by the insertion of a “PRRA” sequence between the S1 and S2 subunits of the SARS-CoV-2 spike protein—absent in closely related betacoronaviruses such as SARS-CoV and RaTG13—has been identified. This motif creates a cleavage site recognized by host proteases like furin, which specifically targets basic amino acid sequences (R-X-X-R). The presence of this motif in SARS-CoV-2 is suggested to contribute to its high infectivity [24]. Furin-mediated cleavage of the spike protein triggers two critical processes:

- Enhanced Receptor Affinity: Cleaved spike protein exhibits higher binding affinity to ACE2, facilitating viral entry.
- Membrane Fusion Facilitation: Exposure of the S2 fusion peptide accelerates viral and host membrane fusion.

These mechanisms may explain SARS-CoV-2′s higher transmissibility compared to SARS-CoV. Furin’s widespread expression in multiple tissues beyond the lungs could account for SARS-CoV-2′s multisystemic symptoms (e.g., gastrointestinal, cardiovascular). The absence of this furin cleavage site in closely related viruses (e.g., SARS-CoV, RaTG13) [24] suggests its critical role in zoonotic adaptation.

Furin plays a central role in SARS-CoV-2 pathogenesis, and its inhibition could block viral entry. Given its involvement in both physiological and pathological processes (infections, cancer, inflammatory diseases), furin is a valuable therapeutic target. A deeper understanding of its molecular mechanisms will aid in developing novel treatments [13,24].

### 3.1. Furin Inhibitors: Challenges and Natural Alternatives

Furin inhibitors must be developed with caution to avoid disrupting physiological functions. Chloromethylketone derivatives and synthetic peptide inhibitors, despite their potential, are unsuitable for clinical use due to high toxicity. However, molecular docking, in silico, and in vitro studies have identified natural compounds with furin inhibitory properties.

The following are examples of such compounds:

- Yang et al. demonstrated that fucoidans (sulfated polysaccharides from brown seaweed) inhibit SARS-CoV-2 entry by targeting both the spike protein and furin [25].
- Olive leaf extract suppressed furin expression in HT-29 colon adenocarcinoma cells [26].
- Omotuyi et al. identified apigenin and quercetin from Aframomum melegueta as furin inhibitors [17].
- Zothanluanga et al. found that isovitexin from Acacia pennata exhibits high binding affinity to furin [18].
- Bandyopadhyay et al. screened 521 phytochemicals and highlighted ochnaflavone (*Lonicera japonica*) and licoflavone B (*Glycyrrhiza glabra*) as potent furin inhibitors [19].

### 3.2. Study Findings: Phenolic Compounds and Furin Inhibition

This study investigated the phenolic profiles and furin inhibition potential of extracts (hexane, chloroform, ethyl acetate) from the *Lamiaceae* and *Asteraceae* family plants, including *Origanum vulgare*, *Mentha piperita*, *Mentha spicata*, *Salvia officinalis*, *Thymus vulgaris*, and *Silybum marianum*. The key findings include the following:

1. Epicatechin showed a statistically significant positive effect on furin inhibition (β = +12.3, *p* < 0.01).
2. Naringenin exhibited a negative effect (β = –8.7, *p* < 0.05), possibly due to allosteric interference with furin’s catalytic [20].
3. Quercetin did not show a significant correlation (*p* > 0.05).
4. Synergistic Effects: Combinations of rutin and epicatechin significantly enhanced inhibition (β = +9.1, *p* < 0.05). Multi-component formulations showed a strong positive correlation with inhibition rates (r = +0.89, *p* < 0.01), with triple combinations yielding 33% higher inhibition than single compounds (*p* < 0.001).

Flavonoids, which contain multiple hydroxyl groups and planar aromatic rings, are known to interact with proteases through hydrogen bonding and π–π stacking interactions with active site residues. Hydroxycinnamic acids (e.g., cinnamic acid, *p*-coumaric acid) may exert weaker inhibitory effects due to their smaller size and lower number of hydroxyl groups, which could limit their binding affinity. Glycosylated flavonoids (e.g., rutin) may show altered activity due to reduced membrane permeability and steric hindrance at the active site. Although direct SAR data for furin is scarce in the literature, our results suggest that polyhydroxylated flavonoids may contribute more significantly to inhibition compared to simpler phenolic acids.

### 3.3. Solvent Effects on Extraction and Bioactivity

- Chloroform: Extracted a diverse range of phenolics (e.g., rutin, epicatechin, sinapic acid), yielding the highest inhibition (e.g., *Origanum vulgare* chloroform extract: 97.44% inhibition).
- Ethyl Acetate: Selectively extracted apigenin-7-O-glucoside and *p*-coumaric acid but also naringenin, which reduced efficacy (*Mentha spicata*: 56.41% inhibition despite high naringenin content).
- Hexane: Limited to lipophilic compounds (e.g., quercetin), resulting in lower inhibition.

Phenolic combinations, particularly epicatechin and rutin, demonstrate synergistic furin inhibition. Chloroform is optimal for extracting bioactive phenolics. These findings align with studies showing that natural multi-component formulations (e.g., MixV: vitamins, polyphenols) achieve higher inhibition than single agents [21,27]. Future research should explore optimized phenolic blends for therapeutic applications.

### 3.4. Limitations of Study

Although the present study offers valuable insights into the phenolic composition and furin protease inhibitory potential of selected Lamiaceae and Asteraceae plant extracts, certain limitations should be acknowledged. Due to the unavailability of high-purity reference standards at the time of analysis, some phenolic compounds such as rosmarinic acid and luteolin, which are commonly found in Lamiaceae species according to existing phytochemical data, were not included in the quantitative analysis. The absence of these compounds may have led to an incomplete representation of the overall phenolic profile of the extracts. Apart from the positive control (chloromethyl ketone, CMK), individual pure phenolic standards were not assayed for furin inhibition in isolation in this study. The focus was on extract-level activities and their correlation with phenolic profiles. Future work will include targeted single-compound assays under identical conditions to directly compare their inhibitory potential with that of the extracts. Despite these limitations, this study is, to our knowledge, the first to evaluate furin protease inhibition using phenolic-rich extracts from Thymus vulgaris, Origanum vulgare, Salvia officinalis, Mentha spicata, Mentha piperita, and Silybum marianum, thus offering a meaningful contribution to the natural product and enzymology literature.

## 4. Materials and Methods

### 4.1. Plant Material

Six medicinal plant species from the families *Lamiaceae* (*Origanum vulgare*, *Thymus vulgaris*, *Mentha piperita*, *Mentha spicata*, Salvia officinalis) and *Asteraceae* (*Silybum marianum*) were selected based on their traditional uses and reported phenolic content. While *Silybum marianum* was purchased from a certified herbal supplier in Turkiye, the remaining species were cultivated under controlled agronomic conditions at the Faculty of Agriculture, Suleyman Demirel University. All plant materials were authenticated based on morphological features by expert taxonomists from the Department of Botany, and representative specimens were retained for reference. Due to commercial sourcing and institutional cultivation, no herbarium voucher numbers were assigned; however, authenticated samples are available upon request.

The plants were harvested at the flowering stage (2–4 months after planting, depending on species). The aerial parts (leaves and flowers) were used for all species except *Silybum marianum*, where seeds were used. Plant materials were cleaned manually to remove soil and debris, washed briefly with distilled water, and dried in a shaded, well-ventilated room at ambient temperature (22–25 °C) until constant weight. Dried plant materials were milled to a fine powder (~0.5 mm mesh) before extraction.

### 4.2. Extraction Procedure

To preserve the distinct phytochemical profiles associated with different polarities, plant samples were extracted separately using three solvents with increasing polarity: hexane, chloroform, and ethyl acetate. Sequential extraction was intentionally avoided.

For each plant species, 20 g of dried aerial parts were macerated in 350 mL of the selected solvent. Extraction was performed using an orbital shaker with an orbital radius of 10 mm at a set speed of 150 rpm for 2 h at room temperature (22–25 °C), followed by 24 h of static maceration. The extraction process involved 2 h of orbital shaking at room temperature, followed by 24 h of static maceration. After extraction, the mixtures were filtered through Whatman No. 1 cellulose filter paper (pore size ~11 µm), and the solvents were evaporated under reduced pressure using a rotary evaporator (Heidolph Hei-VAP, Germany). The resulting crude extracts were then stored at −20 °C until further analysis.

#### 4.2.1. HPLC Analysis and Method Validation

The amounts of phenolic compounds in the plant extracts were determined using a Shimadzu Nexera-i LC-2040C 3D Plus model HPLC system equipped with a photodiode array (PDA) detector (see Supplementary Figures S1–S13). A GL Sciences InterSustain C6 reversed-phase column (Phenyl-Hexyl, 3 μm, 4.6 mm × 150 mm) was used for the analysis. The mobile phase consisted of acetonitrile (Mobile Phase B) and water containing 0.1% formic acid (Mobile Phase A). The column temperature was maintained at 30 °C, and the mobile phase flow rate was set at 1 mL/min (Table 5).

Stock solutions of 1 mg/mL were prepared for each plant extract and phenolic standard compound (quercetin, cinnamic acid, rutin, naringenin, epicatechin, apigenin-7-O-glucoside, and *p*-coumaric acid; all purchased from Sigma-Aldrich/Merck, Darmstadt, Germany; purity ≥ 98%). Calibration curves were constructed using standard solutions at various concentrations. The concentrations of phenolic compounds in the extracts were quantitatively determined based on these calibration curves.

A total of 16 different phenolic compounds were analyzed in the study: 4-hydroxybenzoic acid, rutin, quercetin, gallic acid, chlorogenic acid, caffeic acid, ferulic acid, cichoric acid, *p*-coumaric acid, cinnamic acid, vanillic acid, apigenin-7-O-glucoside, salicylic acid, epicatechin, naringenin, and eriocitrin. Calibration curves were generated with five concentration levels, and quantification was performed at compound-specific wavelengths (λ = 254–360 nm). The content of each phenolic compound in the extracts was calculated and expressed as mg/g of extract. All assays included solvent controls (1× assay buffer and DMSO at the same concentration used to dissolve test samples). Enzyme activity was normalized to the DMSO control, which was defined as 0% inhibition. Solvent controls consistently showed <1% inhibition, confirming no interference with the assay.

Limit of Detection (LOD) and Limit of Quantification (LOQ) were calculated using signal-to-noise ratios of 3:1 and 10:1, respectively. LOD values ranged from 0.005 to 0.020 µg/mL. The method was adapted from previously validated protocols [28,29,30]. 

#### 4.2.2. Furin Inhibition Assay

**Figure 1:**
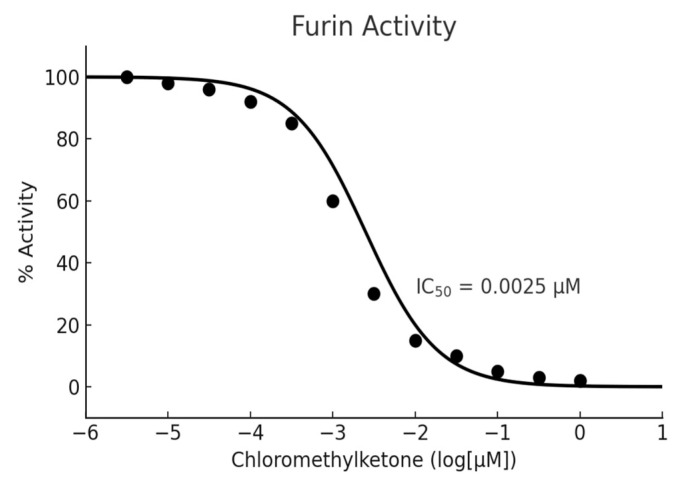
Dose–response curve illustrating the inhibitory effect of chloromethyl ketone on furin protease activity, adapted from the BPS Bioscience user manual. Enzyme activity (% Activity) was plotted against the logarithmic concentration of the compound, with data points representing mean values from replicate experiments (BPS Bioscience, Furin Inhibitor Screening Assay Kit, User Manual).

Furin protease activity was measured using a commercial fluorometric assay kit (Furin Protease Assay Kit #78040, BPS Bioscience, San Diego, CA, USA), following the manufacturer’s instructions. The recombinant human furin used in the assay was the soluble form (amino acids 108–715), supplied by BPS Bioscience, and diluted to 0.5 ng/µL in the assay buffer provided by the manufacturer (proprietary composition). Each well received 10 µL of enzyme solution, corresponding to 5 ng of furin. Chloromethyl ketone (CMK) served as the positive control at 0.5 µM, and DMSO was used as a solvent control (Figure 1).

Briefly, 10 µL of test extract (diluted in 1X assay buffer or 10% DMSO) was added to each well of a 96-well plate, followed by 10 µL of recombinant human furin enzyme. After 5 min, 40 µL of furin-specific fluorogenic substrate (final concentration 2 µM) was added. The substrate was the fluorogenic peptide supplied in the kit (500 µM stock, diluted to 2 µM final concentration), specific for furin cleavage. The reaction was incubated at room temperature for 30 min. Fluorescence intensity was measured using a BioTek Synergy NEO2 plate reader (BioTek, Winooski, VT, USA) (excitation 380 nm, emission 460 nm). Blank values were subtracted from all readings. Dose–response inhibition of CMK was verified using the standard inhibition curve provided by BPS Bioscience, which reports an IC_50_ value of 0.0025 µM; our experimental results were consistent with this value, confirming assay reliability.

### 4.3. Statistical Analysis

All experiments were conducted in triplicate. Data were analyzed using SPSS v25.0 and GraphPad Prism v9.0. Normality was assessed via the Shapiro–Wilk test and homogeneity of variance via Levene’s test. Depending on data distribution, parametric (one-way ANOVA, Tukey’s HSD) or non-parametric (Kruskal–Wallis, Mann–Whitney U) tests were used.

Pearson’s correlation was calculated to assess the relationship between phenolic concentrations and % inhibition. Multiple linear regression was used to model synergistic and antagonistic effects. Additionally, combinatory interactions were assessed using the Bliss Independence Model and Chou–Talalay Combination Index as described in the literature [31,32]. Phenolic combinations were categorized into four groups, single, double, triple, and quadruple components, based on the number of compounds present above LOQ.

## 5. Conclusions

This study confirms the therapeutic potential of phenolic-rich extracts from *Origanum vulgare*, *Salvia officinalis*, *Mentha spicata*, and other medicinal plants as natural furin inhibitors. Statistical analysis revealed that epicatechin significantly enhances furin inhibition, while naringenin exerts an antagonistic effect. Quercetin, though ineffective alone, showed synergistic activity when combined with rutin and epicatechin, highlighting the critical role of compound synergy.

A strong correlation between phenolic diversity and inhibition efficacy (r = +0.89, *p* < 0.01) underscores the value of multi-target phytochemical formulations. Solvent polarity also proved decisive; chloroform extracts achieved the highest inhibition by efficiently extracting both flavonoids and phenolic acids.

These findings emphasize that synergistic phenolic blends, when combined with optimal extraction strategies, offer a promising, low-toxicity alternative to synthetic furin inhibitors. Future research should explore in vivo validation, pharmacokinetics, and nanodelivery systems to fully realize their clinical potential in managing viral infections, cancer, and inflammatory diseases.

## Figures and Tables

**Table 1:** **1** Quantification and furin inhibition of plant extracts.

Plant	Solvent	Quercetin (mg/g Extract)	Rutin (mg/g Extract)	Epicatechin (mg/g Extract)	Cinnamic Acid (mg/g Extract)	Naringenin (mg/g Extract)	Apigenin-7-O-glucoside (mg/g Extract)	Coumaric Acid (mg/g Extract)	% Inhibition	Assumed SD	Mean ± SD
*O. vulgare*	Hexane	0.568	<LOD	<LOD	<LOD	<LOD	<LOD	<LOD	79.49	5.56	79.487 ± 5.564
*O. vulgare*	Chloroform	<LOD	0.051	2.28	0.02	<LOD	<LOD	<LOD	97.44	6.82	97.436 ± 6.821
*M. piperita*	Hexane	0.34	<LOD	<LOD	0.09	<LOD	<LOD	<LOD	84.62	5.92	84.615 ± 5.923
*M. piperita*	Chloroform	<LOD	0.413	<LOD	0.07	8.58	<LOD	<LOD	66.66	4.67	66.66 ± 4.666
*M. spicata*	Hexane	0.233	<LOD	<LOD	0.07	<LOD	<LOD	<LOD	92.30	6.46	92.3 ± 6.461
*M. spicata*	Chloroform	0.061	<LOD	<LOD	<LOD	16.29	<LOD	<LOD	56.41	3.95	56.41 ± 3.949
*M. spicata*	E. Acetate	0.07	1.56	<LOD	2.51	5.32	<LOD	<LOD	97.44	6.82	97.435 ± 6.82
*S. officinalis*	Hexane	0.084	<LOD	<LOD	<LOD	<LOD	<LOD	<LOD	61.54	4.31	61.538 ± 4.308
*S. officinalis*	Chloroform	<LOD	0.23	<LOD	0.02	<LOD	<LOD	<LOD	97.43	6.82	97.434 ± 6.82
*S. officinalis*	E. Acetate	<LOD	<LOD	<LOD	<LOD	<LOD	0.46	0.06	92.31	6.46	92.307 ± 6.461
*T. vulgaris*	Chloroform	<LOD	<LOD	<LOD	0.52	<LOD	<LOD	<LOD	89.74	6.28	89.743 ± 6.282
*T. vulgaris*	E. Acetate	0.289	<LOD	<LOD	0.56	<LOD	0.05	<LOD	56.41	3.95	56.41 ± 3.949
*S. marianum*	Hexane	0.165	<LOD	<LOD	<LOD	<LOD	<LOD	<LOD	74.36	5.21	74.362 ± 5.205

Values are expressed as mean ± standard deviation (SD) of three independent experiments. (*n* = 3), each performed in duplicate.

**Table 2:** Correlations Between Phenolic Compounds And Furin Inhibition.

Compound	Correlation (r)	*p*-Value
Epicatechin	+0.65	<0.01
Cinnamic Acid	+0.48	<0.05
Naringenin	−0.72	<0.001
Quercetin	+0.12	>0.05

**Table 3:** Furin inhibition percentages and phenolic profiles of plant extracts according to combination types.

Combination Type	Plant Extract	Components	Number of Samples	Average Furin (%) Inhibition
	*Origanum vulgare* (Hexane)	Quercetin		
Single	*Mentha spicata* (Hexane)	Quercetin	5	73.2 ± 3.1
	*Salvia officinalis* (Hexane)	Quercetin		
	*Silybum marianum* (Hexane)	Quercetin		
Double	*Salvia officinalis* (Chloroform)	Rutin + Cinnamic Acid	3	85.1 ± 2.8
	*Mentha piperita* (Chloroform)	Rutin + Naringenin		
Triple	*Origanum vulgare* (Chloroform)	Rutin + Epichatechin + Cinnamic Acid	2	97.4 ± 0.1
	*Thymus vulgaris* (Chloroform)	Quercetin + Cinnamic Acid + Epichatechin		
Quadruple	*Mentha spicata* (Ethyl Acetate)	Quercetin + Rutin + Cinnamic Acid + Naringenin	1	97.4 ± 0.1

**Table 4:** Effect of solvent polarity on phenolic compound diversity.

Solvent	Extracted Phenolic Compounds	Plant Species
Chloroform	Rutin, Epicatechin, Cinnamic Acid	*Origanum vulgare*, *Salvia officinalis*
Ethyl Acetate	Apigenin-7-O-glucoside, *p*-Coumaric Acid, Naringenin	*Mentha spicata*, *Salvia officinalis*
Hexane	Quercetin, Cinnamic Acid (limited)	*Origanum vulgare*, *Mentha spicata*

**Table 5:** Chromatographic separation conditions for quantification of phenolic compounds.

Parameter	Condition/Value
Instrument	Shimadzu Nexera-i LC-2040C 3D Plus
Detector	Photodiode Array (PDA)
Column	GL Sciences InterSustain C6 (Phenyl-Hexyl), 3 µm, 4.6 × 150 mm
Column Temperature	30 °C
Mobile Phase A	Water with 0.1% formic acid
Mobile Phase B	Acetonitrile
Elution Mode	Gradient
Flow Rate	1.0 mL/min
Injection Volume	20 µL
Detection Wavelengths (λmax)	Compound-specific (254–360 nm)
Run Time	~35 min (depending on gradient program)
LOD/LOQ	LOD: 0.015–0.045 µg/mL; LOQ: 0.050–0.130 µg/mL
Quantification Method	External calibration with 5 concentration levels
Software	Shimadzu LabSolutions LC v5.97 (DB)

## Data Availability

The raw data supporting the conclusions of this article will be made available by the authors on request, without undue reservation.

## Supplementary Materials

The following supporting information can be downloaded at: https://www.mdpi.com/article/10.3390/molecules30173450/s1, Figure S1: HPLC chromatogram of Origanum vulgare hexane extract; Figure S2: HPLC chromatogram of Origanum vulgare chloroform extract; Figure S3: HPLC chromatogram of Mentha piperita hexane extract; Figure S4: HPLC chromatogram of Mentha piperita chloroform extract; Figure S5: HPLC chromatogram of Mentha spicata hexane extract; Figure S6: HPLC chromatogram of Mentha spicata chloroform extract; Figure S7: HPLC chromatogram of Mentha spicata ethyl acetate extract; Figure S8: HPLC chromatogram of Salvia officinalis hexane extract; Figure S9: HPLC chromatogram of Salvia officinalis chloroform extract; Figure S10: HPLC chromatogram of Salvia officinalis ethyl acetate extract; Figure S11: HPLC chromatogram of Thymus vulgaris chloroform extract; Figure S12: HPLC chromatogram of Thymus vulgaris ethyl acetate extract; Figure S13: HPLC chromatogram of Silybum marianum hexane extract.

## Abbreviations

The following abbreviations are used in this manuscript:

ACE2	Angiotensin-Converting Enzyme 2
ANOVA	Analysis of Variance
BAP	Scientific Research Project
CMK	Chloromethyl Ketone
COVID-19	Coronavirus Disease 2019
DMSO	Dimethyl Sulfoxide
HA	Hemagglutinin (in influenza virus)
HPLC	High-Performance Liquid Chromatography
IC_50_	Half-Maximal Inhibitory Concentration
IGF	Insulin-like Growth Factor
LOD	Limit of Detection
LOQ	Limit of Quantification
MMPs	Matrix Metalloproteinases
PDGF	Platelet-Derived Growth Factor
RAAS	Renin–Angiotensin–Aldosterone System
r	Pearson Correlation Coefficient
SARS-CoV-2	Severe Acute Respiratory Syndrome Coronavirus 2
TGF-β	Transforming Growth Factor Beta
VEGF	Vascular Endothelial Growth Factor
