# Supplementary material for: Synergistic Phenolic Compounds in Medicinal Plant Extracts: Enhanced Furin Protease Inhibition via Solvent-Specific Extraction from Lamiaceae and Asteraceae Families
Source: Molecules. 2025 Aug 22;30(17):3450. doi: 10.3390/molecules30173450 (PMC12430539; doi:10.3390/molecules30173450)
Supplement: Supplementary file 1 [file molecules-30-03450-s001.zip › molecules-3790502-supplementary.pdf]

Figure S1

HPLC Results of Origanum vulgare Hexane Extract

SHIMADZU LabSolutions **Analysis Report**

<Sample Information>

Sample Name	: 1_2000ppm		
Sample ID	:		
Data Filename	: 1_2000ppm.lcd		
Method Filename	: 15 fenolik madde çalışması 36-kalibrasyon ok.lcm		
Batch Filename	: 6 örnek.lcb		
Vial #	: 3-1	Sample Type	: Unknown
Injection Volume	: 10 uL		
Date Acquired	: 1.10.2024 12:22:02	Acquired by	: System Administrator
Date Processed	: 7.10.2024 13:07:54	Processed by	: System Administrator

<Chromatogram>

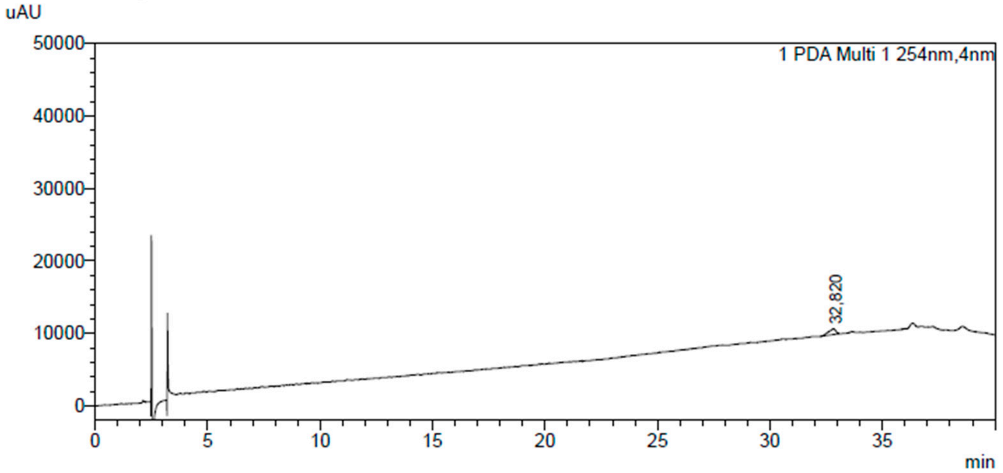

<Peak Table>

PDA Ch1 254nm

Peak#	Ret. Time	Area	Height	Conc.	Unit	Mark	Name
1	32,820	17638	778	0,568	mg/L		Quercetin
Total		17638	778				

Figure S2

HPLC Results of Origanum vulgare Chloroform Extract

SHIMADZU LabSolutions

Analysis Report

<Sample Information>

Sample Name	: 2_1000ppm		
Sample ID	:		
Data Filename	: 2_1000ppm.lcd		
Method Filename	: 15 fenolik madde çalışması 36-kalibrasyon ok.lcm		
Batch Filename	: 7 örnek.lcb		
Vial #	: 3-5	Sample Type	: Unknown
Injection Volume	: 10 uL		
Date Acquired	: 30.09.2024 17:28:20	Acquired by	: System Administrator
Date Processed	: 7.10.2024 12:57:39	Processed by	: System Administrator

<Chromatogram>

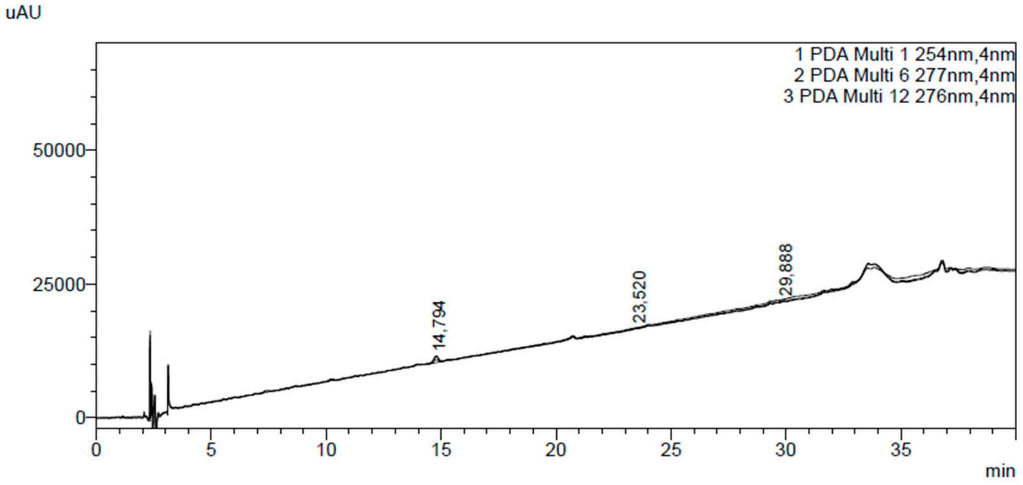

<Peak Table>

PDA Ch1 254nm

Peak#	Ret. Time	Area	Height	Conc.	Unit	Mark	Name
1	23,520	1034	49	0,051	mg/L		Rutin
Total		1034	49				

PDA Ch6 277nm

Peak#	Ret. Time	Area	Height	Conc.	Unit	Mark	Name
1	14,794	16287	1162	2,277	mg/L		Epicatechin
Total		16287	1162				

PDA Ch12 276nm

Peak#	Ret. Time	Area	Height	Conc.	Unit	Mark	Name
1	29,888	2031	196	0,023	mg/L		Cinnamic Acid
Total		2031	196				

Figure S3

HPLC Results of *Mentha   pipperita* Hexane Extract

SHIMADZU LabSolutions **Analysis Report**

<Sample Information>

Sample Name	: 4_2000ppm		
Sample ID	:		
Data Filename	: 4_2000ppm.lcd		
Method Filename	: 15 fenolik madde çalışması 36-kalibrasyon ok.lcm		
Batch Filename	: 6 örnek.lcb		
Vial #	: 3-2	Sample Type	: Unknown
Injection Volume	: 10 uL		
Date Acquired	: 1.10.2024 13:02:25	Acquired by	: System Administrator
Date Processed	: 7.10.2024 12:54:26	Processed by	: System Administrator

<Chromatogram>

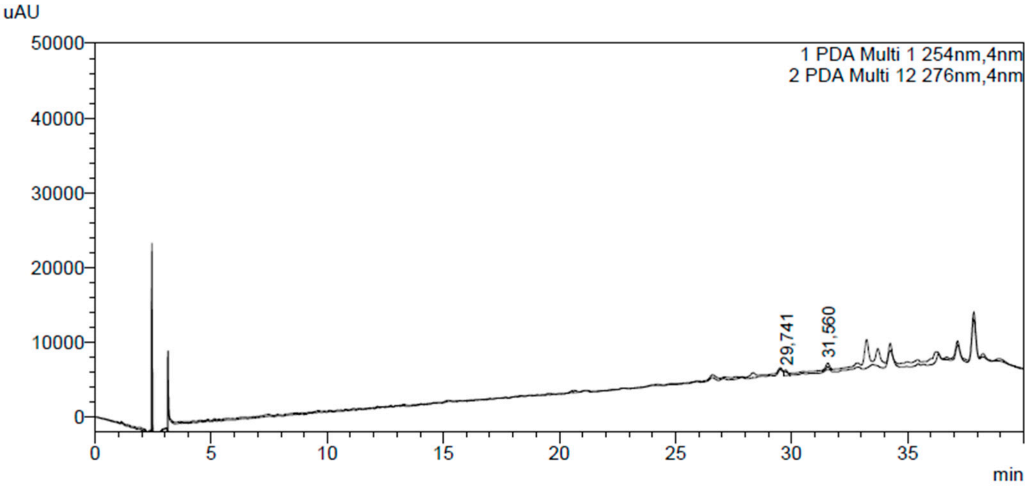

<Peak Table>

PDA Ch1 254nm

Peak#	Ret. Time	Area	Height	Conc.	Unit	Mark	Name
1	31,560	10671	896	0,344	mg/L		Quercetin
Total		10671	896				

PDA Ch12 276nm

Peak#	Ret. Time	Area	Height	Conc.	Unit	Mark	Name
1	29,741	7928	743	0,089	mg/L		Cinnamic Acid
Total		7928	743				

Figure S4

HPLC Results of *Mentha Piperita* Chloroform extract

SHIMADZU LabSolutions **Analysis Report**

<Sample Information>

Sample Name	: 5_1000ppm		
Sample ID	:		
Data Filename	: 5_1000ppm.lcd		
Method Filename	: 15 fenolik madde çalışması 36-kalibrasyon ok.lcm		
Batch Filename	: 7 örnek.lcb		
Vial #	: 3-7	Sample Type	: Unknown
Injection Volume	: 10 uL		
Date Acquired	: 30.09.2024 18:49:03	Acquired by	: System Administrator
Date Processed	: 7.10.2024 12:44:44	Processed by	: System Administrator

<Chromatogram>

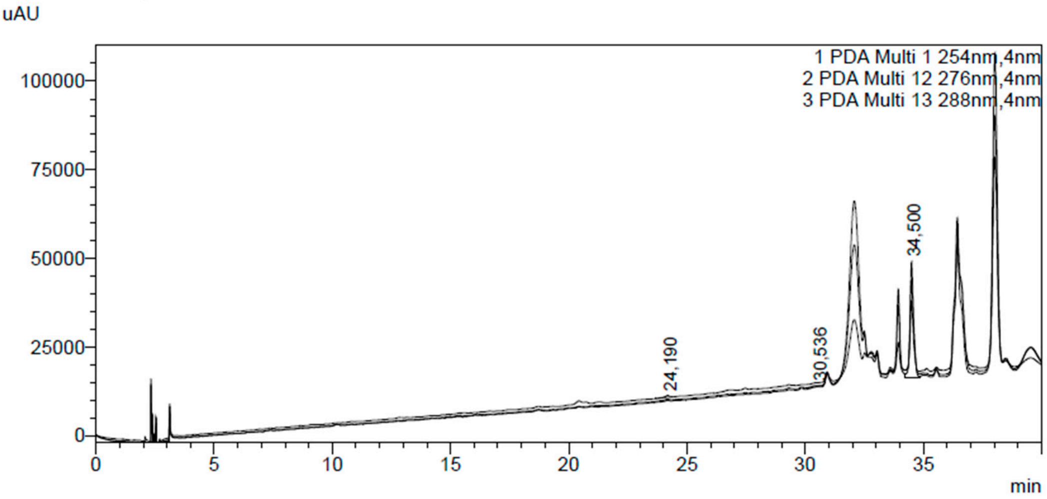

<Peak Table>

PDA Ch1 254nm

Peak#	Ret. Time	Area	Height	Conc.	Unit	Mark	Name
1	24,190	8392	569	0,413	mg/L		Rutin
Total		8392	569				

PDA Ch12 276nm

Peak#	Ret. Time	Area	Height	Conc.	Unit	Mark	Name
1	30,536	6245	214	0,070	mg/L		Cinnamic Acid
Total		6245	214				

PDA Ch13 288nm

Peak#	Ret. Time	Area	Height	Conc.	Unit	Mark	Name
1	34,500	346837	32579	8,580	mg/L		Naringenin
Total		346837	32579				

Figure S5

HPLC Results of *Mentha Spicata* Hexane extract

SHIMADZU LabSolutions **Analysis Report**

<Sample Information>

Sample Name	: 7_2000ppm		
Sample ID	:		
Data Filename	: 7_2000ppm.lcd		
Method Filename	: 15 fenolik madde çalışması 36-kalibrasyon ok.lcm		
Batch Filename	: 6 örnek.lcb		
Vial #	: 3-3	Sample Type	: Unknown
Injection Volume	: 10 uL		
Date Acquired	: 1.10.2024 13:42:49	Acquired by	: System Administrator
Date Processed	: 7.10.2024 12:39:36	Processed by	: System Administrator

<Chromatogram>

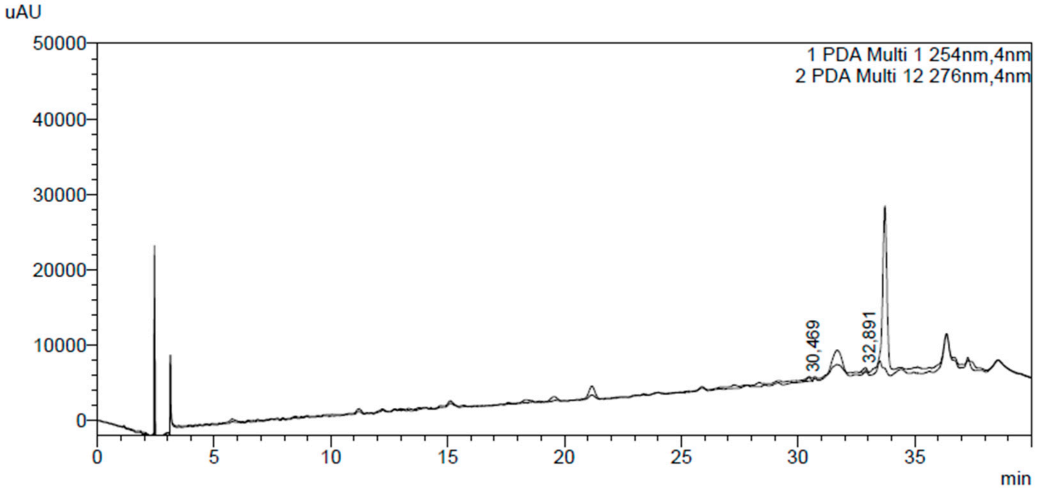

<Peak Table>

PDA Ch1 254nm

Peak#	Ret. Time	Area	Height	Conc.	Unit	Mark	Name
1	32,891	7224	602	0,233	mg/L		Quercetin
Total		7224	602				

PDA Ch12 276nm

Peak#	Ret. Time	Area	Height	Conc.	Unit	Mark	Name
1	30,469	5889	527	0,066	mg/L		Cinnamic Acid
Total		5889	527				

Figure S6

HPLC Results of *Mentha Spicata* Chloroform extract

SHIMADZU LabSolutions

Analysis Report

<Sample Information>

Sample Name	: 8_1000ppm		
Sample ID	:		
Data Filename	: 8_1000ppm.lcd		
Method Filename	: 15 fenolik madde çalışması 36-kalibrasyon ok.lcm		
Batch Filename	: 7 örnek.lcb		
Vial #	: 3-6	Sample Type	: Unknown
Injection Volume	: 10 uL		
Date Acquired	: 30.09.2024 18:08:42	Acquired by	: System Administrator
Date Processed	: 7.10.2024 12:35:18	Processed by	: System Administrator

<Chromatogram>

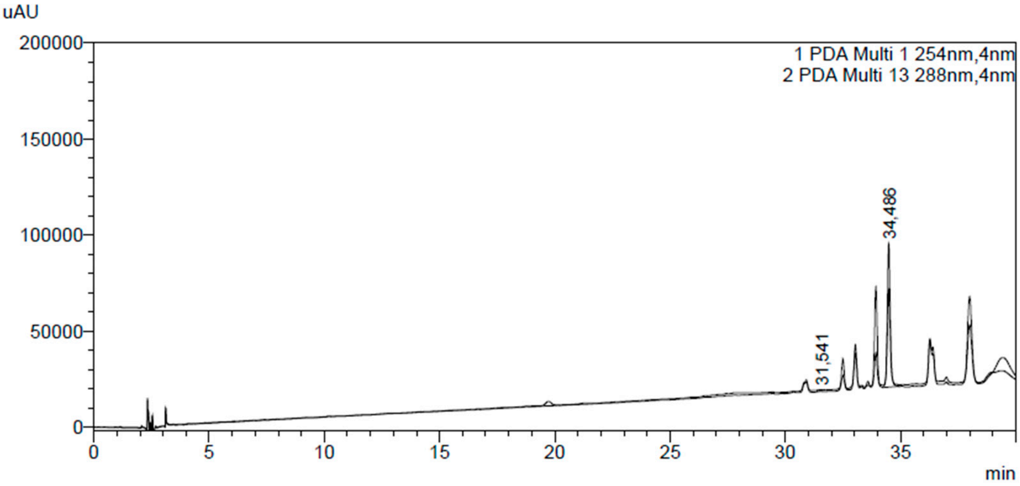

<Peak Table>

PDA Ch1 254nm

Peak#	Ret. Time	Area	Height	Conc.	Unit	Mark	Name
1	31,541	1888	222	0,061	mg/L		Quercetin
Total		1888	222				

PDA Ch13 288nm

Peak#	Ret. Time	Area	Height	Conc.	Unit	Mark	Name
1	34,486	658612	74602	16,292	mg/L		Naringenin
Total		658612	74602				

Figure S7

HPLC Results of *Mentha spicata* Etilasetat extract

SHIMADZU LabSolutions

Analysis Report

<Sample Information>

Sample Name	: 9_1000ppm		
Sample ID	:		
Data Filename	: 9_1000ppm.lcd		
Method Filename	: 15 fenolik madde çalışması 36-kalibrasyon ok.lcm		
Batch Filename	: 5 örnek.lcb		
Vial #	: 1-37	Sample Type	: Unknown
Injection Volume	: 10 uL		
Date Acquired	: 26.09.2024 16:39:32	Acquired by	: System Administrator
Date Processed	: 7.10.2024 12:22:34	Processed by	: System Administrator

<Chromatogram>

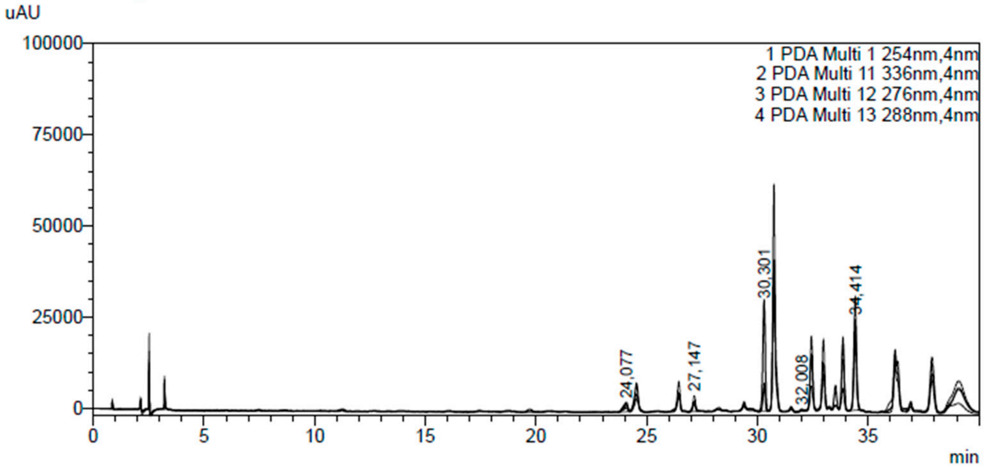

<Peak Table>

PDA Ch1 254nm

Peak#	Ret. Time	Area	Height	Conc.	Unit	Mark	Name
1	24,077	31599	2322	1,555	mg/L		Rutin
2	32,008	2179	323	0,070	mg/L		Quercetin
Total		33778	2645				

PDA Ch11 336nm

Peak#	Ret. Time	Area	Height	Conc.	Unit	Mark	Name
1	27,147	39669	4275	0,870	mg/L		Apigenin-7-O-glucoside
Total		39669	4275				

PDA Ch12 276nm

Peak#	Ret. Time	Area	Height	Conc.	Unit	Mark	Name
1	30,301	223699	29547	2,509	mg/L		Cinnamic Acid
Total		223699	29547				

PDA Ch13 288nm

Peak#	Ret. Time	Area	Height	Conc.	Unit	Mark	Name
1	34,414	214864	24825	5,315	mg/L		Naringenin
Total		214864	24825				

Figure S8

HPLC Results of *Salvia Oficinalis* Hexane extract

SHIMADZU LabSolutions

Analysis Report

<Sample Information>

Sample Name	: 10_2000ppm		
Sample ID	:		
Data Filename	: 10_2000ppm.lcd		
Method Filename	: 15 fenolik madde çalışması 36-kalibrasyon ok.lcm		
Batch Filename	: 6 örnek.lcb		
Vial #	: 3-4	Sample Type	: Unknown
Injection Volume	: 10 uL		
Date Acquired	: 1.10.2024 14:23:11	Acquired by	: System Administrator
Date Processed	: 7.10.2024 14:39:45	Processed by	: System Administrator

<Chromatogram>

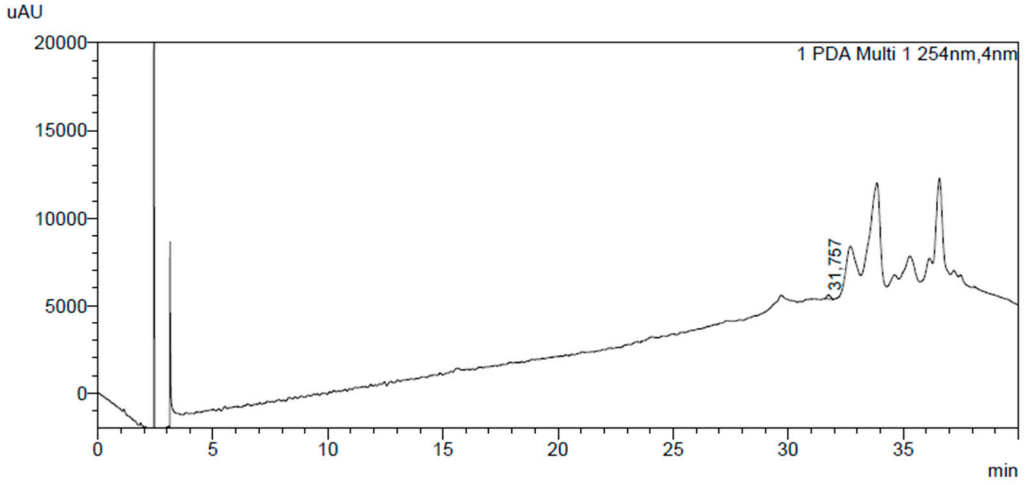

<Peak Table>

PDA Ch1 254nm

Peak#	Ret. Time	Area	Height	Conc.	Unit	Name
1	31,757	2601	232	0,084	mg/L	Quercetin
Total		2601	232			

Figure S9

HPLC Results of *Salvia Oficinalis* Chloroform extract

SHIMADZU LabSolutions

Analysis Report

<Sample Information>

Sample Name	: 11_1000ppm		
Sample ID	:		
Data Filename	: 11_1000ppm.lcd		
Method Filename	: 15 fenolik madde çalışması 36-kalibrasyon ok.lcm		
Batch Filename	: 7 örnek.lcb		
Vial #	: 3-1	Sample Type	: Unknown
Injection Volume	: 10 uL		
Date Acquired	: 30.09.2024 14:46:48	Acquired by	: System Administrator
Date Processed	: 7.10.2024 14:37:42	Processed by	: System Administrator

<Chromatogram>

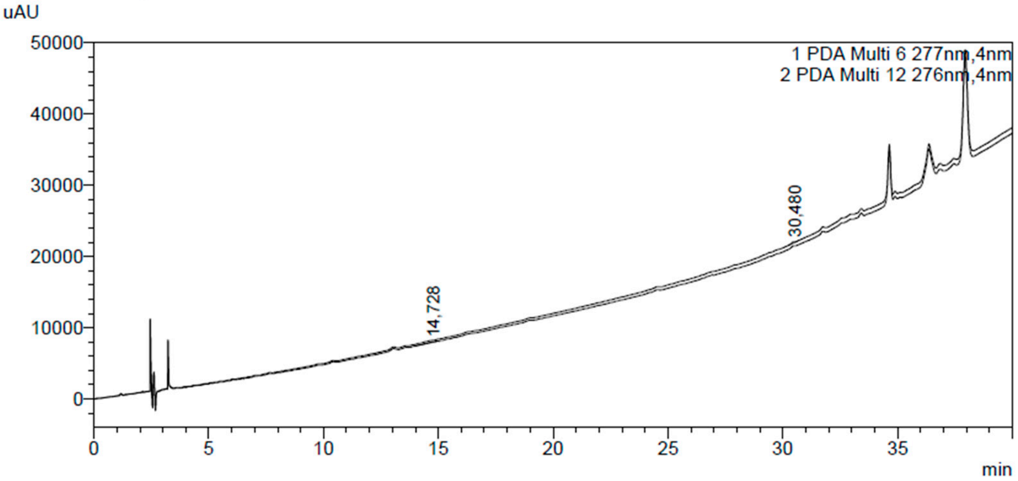

<Peak Table>

PDA Ch6 277nm

Peak#	Ret. Time	Area	Height	Conc.	Unit	Mark	Name
1	14,728	1611	56	0,225	mg/L		Epicatechin
Total		1611	56				

PDA Ch12 276nm

Peak#	Ret. Time	Area	Height	Conc.	Unit	Mark	Name
1	30,480	1878	162	0,021	mg/L		Cinnamic Acid
Total		1878	162				

Figure S10

HPLC Results of *Salvia Oficinalis* Etilasetat extract

SHIMADZU LabSolutions **Analysis Report**

<Sample Information>

Sample Name	: 12_1000ppm		
Sample ID	:		
Data Filename	: 12_1000ppm.lcd		
Method Filename	: 15 fenolik madde çalışması 36-kalibrasyon ok.lcm		
Batch Filename	: 5 örnek.lcb		
Vial #	: 1-38	Sample Type	: Unknown
Injection Volume	: 10 uL		
Date Acquired	: 26.09.2024 17:19:54	Acquired by	: System Administrator
Date Processed	: 7.10.2024 14:35:53	Processed by	: System Administrator

<Chromatogram>

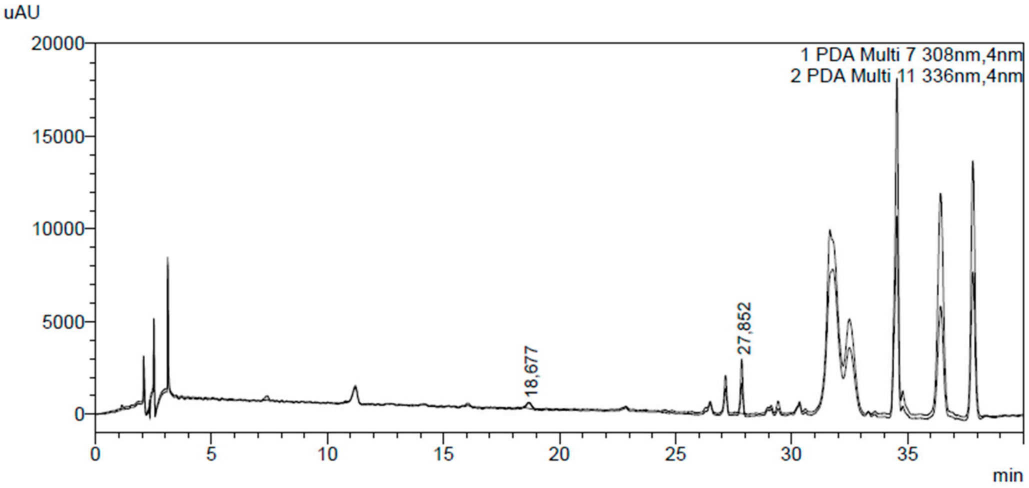

<Peak Table>

PDA Ch7 308nm

Peak#	Ret. Time	Area	Height	Conc.	Unit	Mark	Name
1	18,677	4334	313	0,055	mg/L		p-Coumaric Acid
Total		4334	313				

PDA Ch11 336nm

Peak#	Ret. Time	Area	Height	Conc.	Unit	Mark	Name
1	27,852	20859	2911	0,457	mg/L		Apigenin-7-O-glucoside
Total		20859	2911				

Figure S11

HPLC Results of *Thymus vulgaris* Chloroform extract

SHIMADZU LabSolutions

Analysis Report

<Sample Information>

Sample Name : 14_1000ppm
Sample ID :
Data Filename : 14_1000ppm.lcd
Method Filename : 15 fenolik madde çalışması 36-kalibrasyon ok.lcm
Batch Filename : 7 örnek.lcb
Vial # : 3-2 Sample Type : Unknown
Injection Volume : 10 uL
Date Acquired : 30.09.2024 15:27:11 Acquired by : System Administrator
Date Processed : 7.10.2024 14:32:48 Processed by : System Administrator

<Chromatogram>

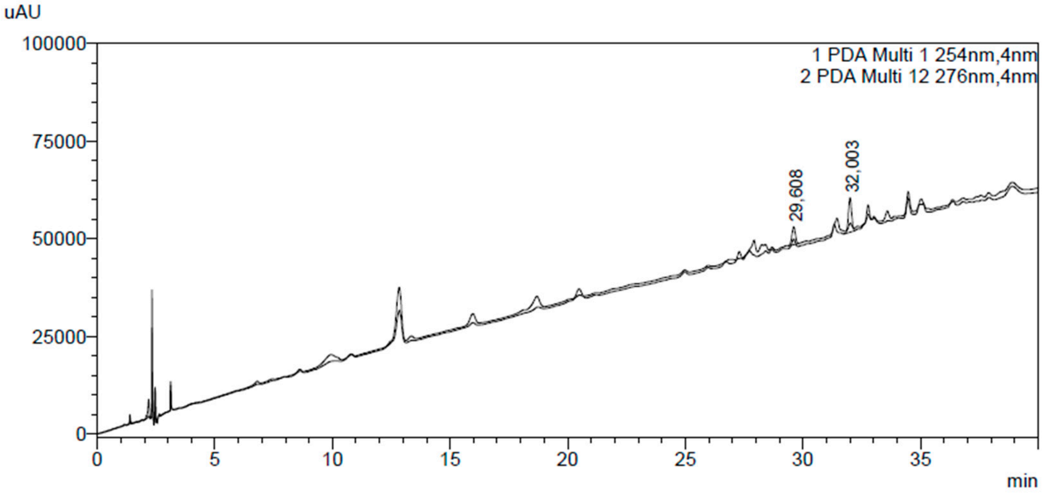

<Peak Table>

PDA Ch1 254nm

Peak#	Ret. Time	Area	Height	Conc.	Unit	Mark	Name
1	32,003	84147	8814	2,710	mg/L		Quercetin
Total		84147	8814				

PDA Ch12 276nm

Peak#	Ret. Time	Area	Height	Conc.	Unit	Mark	Name
1	29,608	46339	4562	0,520	mg/L		Cinnamic Acid
Total		46339	4562				

Figure S12

HPLC Results of *Thymus vulgaris* Etilasetat extract

SHIMADZU LabSolutions **Analysis Report**

<Sample Information>

Sample Name : 15_10000ppm
Sample ID :
Data Filename : 15_1000ppm.lcd
Method Filename : 15 fenolik madde çalışması 36-kalibrasyon ok.lcm
Batch Filename : 5 örnek.lcb
Vial # : 1-39 Sample Type : Unknown
Injection Volume : 10 uL
Date Acquired : 26.09.2024 18:00:18 Acquired by : System Administrator
Date Processed : 7.10.2024 14:29:17 Processed by : System Administrator

<Chromatogram>

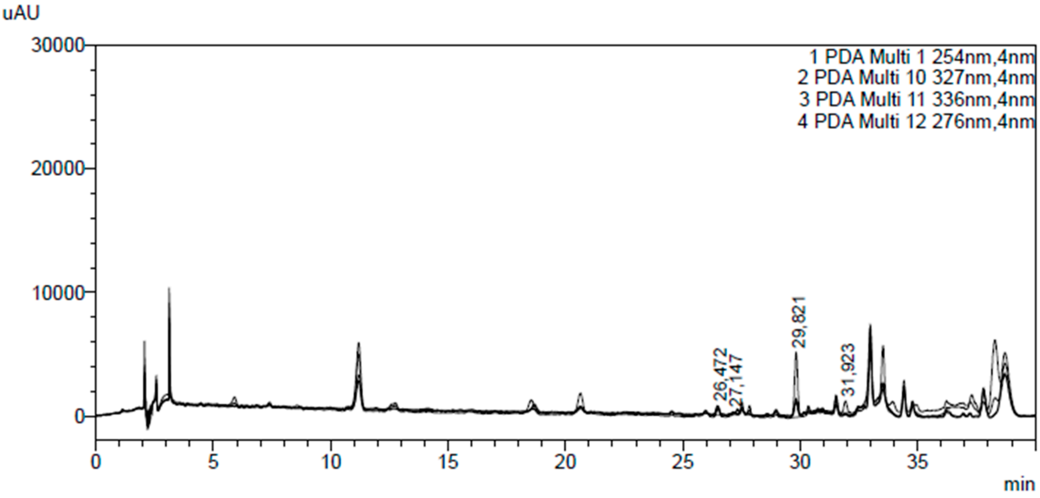

<Peak Table>

PDA Ch1 254nm

Peak#	Ret. Time	Area	Height	Conc.	Unit	Mark	Name
1	31,923	8975	972	0,289	mg/L		Quercetin
Total		8975	972				

PDA Ch10 327nm

Peak#	Ret. Time	Area	Height	Conc.	Unit	Mark	Name
1	26,472	6456	721	0,583	mg/L		Chicoric Acid
Total		6456	721				

PDA Ch11 336nm

Peak#	Ret. Time	Area	Height	Conc.	Unit	Mark	Name
1	27,147	2225	196	0,049	mg/L		Apigenin-7-O-glucoside
Total		2225	196				

PDA Ch12 276nm

Peak#	Ret. Time	Area	Height	Conc.	Unit	Mark	Name
1	29,821	49733	5225	0,558	mg/L		Cinnamic Acid
Total		49733	5225				

Figure S13

HPLC Results of *Silybum Marianum* Hexane extract

SHIMADZU LabSolutions

Analysis Report

<Sample Information>

Sample Name	: 20_4000ppm		
Sample ID	:		
Data Filename	: 20_4000ppm.lcd		
Method Filename	: 15 fenolik madde çalışması 36-kalibrasyon ok.lcm		
Batch Filename	: 6 örnek.lcb		
Vial #	: 3-6	Sample Type	: Unknown
Injection Volume	: 10 uL		
Date Acquired	: 1.10.2024 15:43:57	Acquired by	: System Administrator
Date Processed	: 7.10.2024 13:06:52	Processed by	: System Administrator

<Chromatogram>

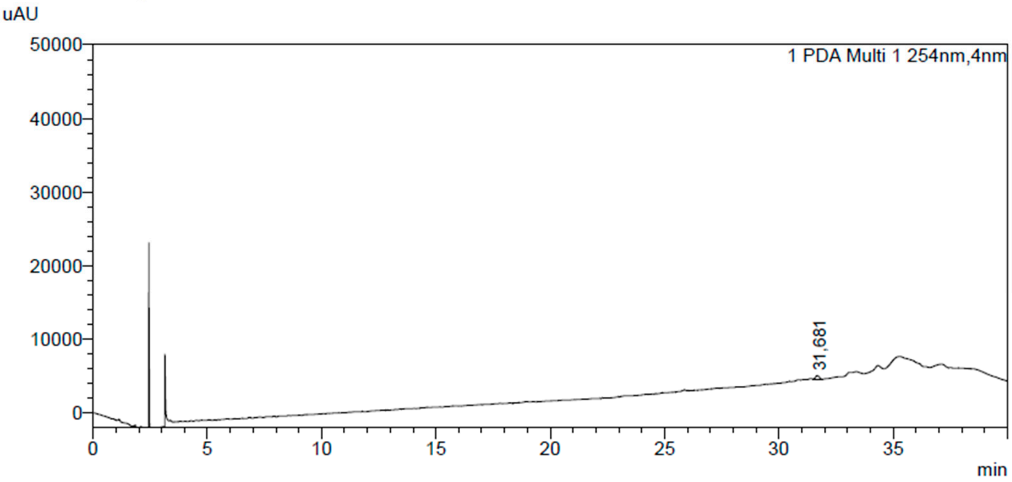

<Peak Table>

PDA Ch1 254nm

Peak#	Ret. Time	Area	Height	Conc.	Unit	Mark	Name
1	31,681	5122	499	0,165	mg/L		Quercetin
Total		5122	499				
